# Isolation and engineering of a *Listeria grayi* bacteriophage

**DOI:** 10.1038/s41598-021-98134-1

**Published:** 2021-09-23

**Authors:** Stephen Erickson, John Paulson, Matthew Brown, Wendy Hahn, Jose Gil, Rocío Barron-Montenegro, Andrea I. Moreno-Switt, Marcia Eisenberg, Minh M. Nguyen

**Affiliations:** 1 Laboratory Corporation of America Holdings, New Brighton, MN 55112 USA; 2 Laboratory Corporation of America Holdings, Burlington, NC 27215 USA; 3 Laboratory Corporation of America Holdings, Los Angeles, CA 90062 USA; 4 Escuela de Medicina Veterinaria, Facultad de Agronomía e Ingeniería Forestal, Facultad de Ciencias Biológicas, Facultad de Medicina, Pontificia Universidad Católica de Chile, Santiago, Chile; 5 Millennium Initiative for Collaborative Research on Bacteria Resistance (MICROB-R), Santiago, Chile

**Keywords:** Bacteriophages, Applied microbiology

## Abstract

The lack of bacteriophages capable of infecting the *Listeria* species, *Listeria grayi*, is academically intriguing and presents an obstacle to the development of bacteriophage-based technologies for *Listeria*. We describe the isolation and engineering of a novel *L. grayi* bacteriophage, LPJP1, isolated from farm silage. With a genome over 200,000 base pairs, LPJP1 is the first and only reported jumbo bacteriophage infecting the *Listeria* genus. Similar to other Gram-positive jumbo phages, LPJP1 appeared to contain modified base pairs, which complicated initial attempts to obtain genomic sequence using standard methods. Following successful sequencing with a modified approach, a recombinant of LPJP1 encoding the NanoLuc luciferase was engineered using homologous recombination. This luciferase reporter bacteriophage successfully detected 100 stationary phase colony forming units of both subspecies of *L. grayi* in four hours. A single log phase colony forming unit was also sufficient for positive detection in the same time period. The recombinant demonstrated complete specificity for this particular *Listeria* species and did not infect 150 non-*L. grayi Listeria* strains nor any other bacterial genus. LPJP1 is believed to be the first reported lytic bacteriophage of *L. grayi* as well as the only jumbo bacteriophage to be successfully engineered into a luciferase reporter.

## Introduction

*Listeria grayi* includes two genetically related subspecies, *L. grayi* subsp. *grayi* and *L. grayi* subsp. *murrayi*. Although typically classified as non-pathogenic, *L. grayi* has been associated with severe disease in rare cases involving immunocompromised individuals^1–3^. Despite lacking clinical significance to the general population, *L. grayi* shares a number of important characteristics with the food-borne pathogen *L. monocytogenes*, including motility and growth at refrigerated temperatures^4^. These shared features have allowed this and other non-pathogenic *Listeria* species to serve as useful tools in food safety. One particular example of this is seen in *Listeria* environmental monitoring programs^5^. Detection of any viable *Listeria* species during sampling leads to preventative intervention to eliminate growth conditions and preempt contamination with pathogenic species, particularly *L. monocytogenes*. Thus, studies on *L. grayi* and other non-pathogenic *Listeria* species are beneficial to both academic understanding and public health initiatives.

To our knowledge, no bacteriophage has been reported to infect and lyse *L. grayi*. This is unexpected as the number of *Listeria* species has grown from six in 1984 to 21 in 2020, and *L. grayi* was described over 50 years ago, providing ample time for phage discovery^4^. In addition, hundreds of *Listeria* phages have been described over the years, and the lack of lytic phages appears to be unique to *L. grayi*. None of 16 phages comprising a *Listeria* typing system could lyse *L. grayi*^6^. Further, isolation of *Listeria*-specific phage from food processing plants found phage capable of lysing all tested *Listeria* species except *L. grayi*^7^. The lack of reported lytic phages for this species could have indicated a unique phage resistance mechanism or imply that such a phage was rare or challenging to isolate. Given the use of *L. monocytogenes* for isolation of phage in these previously described studies, *Listeria* phage specific to *L. grayi* would also remain undetected.

Engineered bacteriophages have great potential as diagnostic tools. The use of engineered recombinant phages encoding a reporter enzyme, such as a luciferase, is particularly promising in facilitating detection of bacteria. When viable hosts are present in a sample, recombinant phages will infect these cells, leading to production of the reporter enzyme and a detectable signal^8^. Phages have been recently engineered in this fashion to detect a variety of bacteria, ranging from food-borne contaminants such as *E. coli* O157:H7, *Salmonella* and *L. monocytogenes* to clinical pathogens such as *S. aureus* and *M. tuberculosis*^9–13^. The viability of phage-based reporters is linked to successful isolation and engineering of suitable phage cocktails^14^. Despite growing phage collections, bacterial species susceptible to limited or no known phages present a challenge to developing this technology across diverse niches.

Luciferase reporter phages have been previously engineered in the pursuit of specific and sensitive detection of *Listeria* species in food products^11,15^. Initial assays utilized a lytic phage, A511, that was engineered to encode the LuxAB luciferase from *Vibrio harveyi*^11^. Among known phages, A511 is an excellent choice for a reporter, as it broadly recognizes the *Listeria* genus, covering most serotypes and species. This method has recently been further improved by incorporating NanoLuc, an engineered luciferase evolved from the *Oplophorus gracilirostris* luciferase^15^. Using NanoLuc-encoding A511 recombinants, reliable detection of a single CFU of *L. monocytogenes* in a 25 g portion of various food products was possible after a 20 h enrichment. Despite the successes and proven viability of this approach, *L. grayi* continues to be absent in the host range of these reporters and a blind spot in phage-based technologies.

The primary objectives of this study were to: (1) isolate a lytic bacteriophage recognizing *L. grayi* and (2) engineer and characterize a NanoLuc-encoding recombinant of said phage. Using silage, a common medium for isolating *Listeria* phages, this report describes the isolation and characterization of the first phage, to our knowledge, uniquely capable of lysing *L. grayi*. Further, homologous recombination was used to create a NanoLuc-encoding recombinant, which ultimately proved capable of sensitive and accurate detection of *L. grayi*, the first in phage-based technologies.

## Results

### Isolation and basic characterization of LPJP1

Silage has been recognized as an excellent source of diverse *Listeria* bacteriophages^16^. Silage obtained from a Wisconsin farm was probed for the presence of phages capable of lysing *L. grayi*. From this material, a virulent phage referred to as LPJP1 was isolated on *L. grayi* lawns and subsequently purified by serial plaque isolation. LPJP1 formed small clear plaques at 30 °C on the *L. grayi* strain ATCC 19,120 (Supplementary Fig. 1). Interestingly, plaques were not observed at 37 °C (data not shown). The mechanism behind this phenotype is not fully understood but is consistent with temperature-dependent plaque formation observed with other *Listeria* phages^17^. A one-step growth curve of LPJP1 revealed a burst size of approximately 50 to 60 pfu/cell with a latent period of roughly 70 min. To our knowledge, LPJP1 is the first published phage capable of lysing and forming plaques on *L. grayi*.

**Figure 1:**
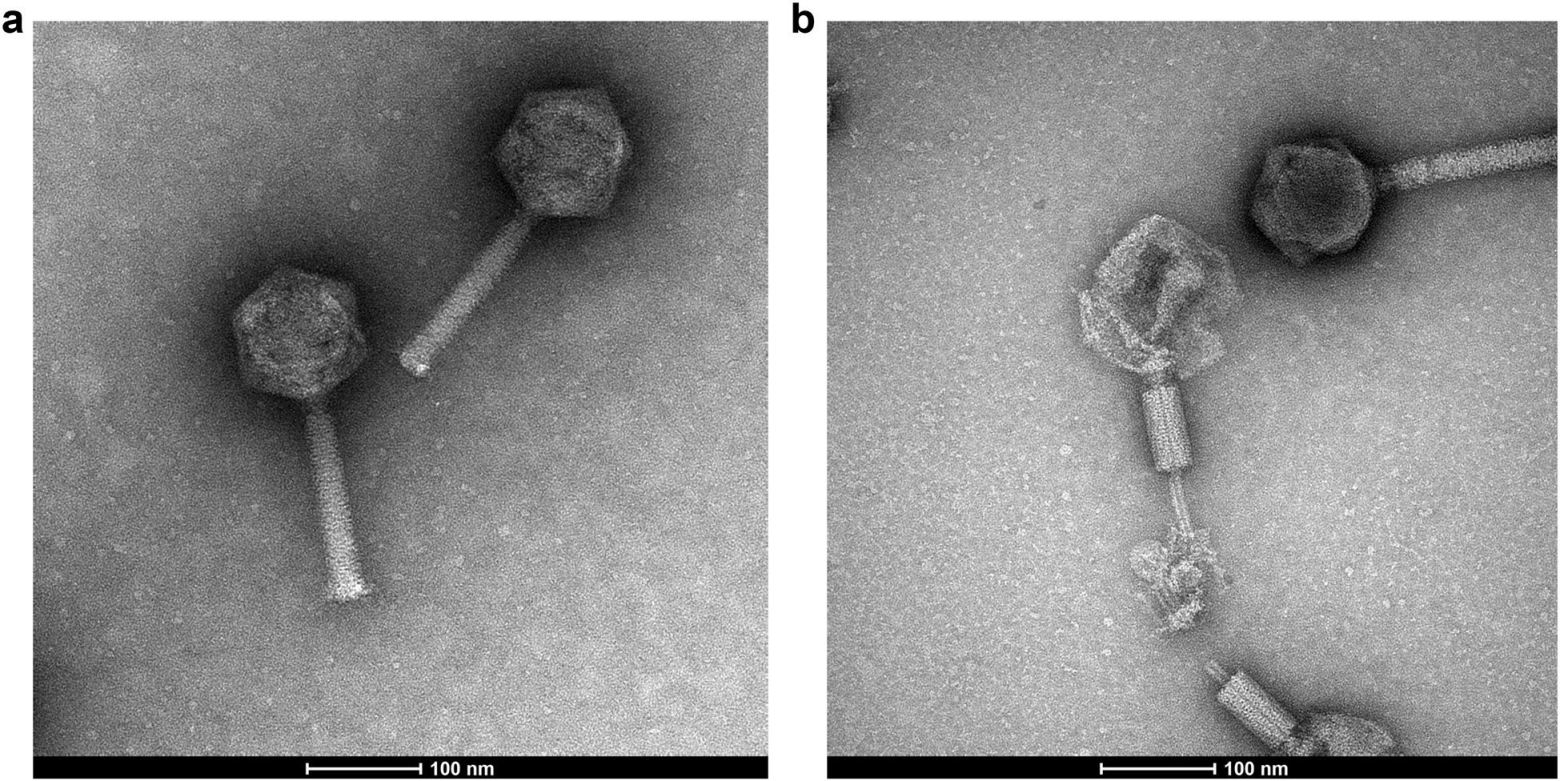
Phage morphology visualized by transmission electron microscopy revealing LPJP1’s (**a**) large icosahedral head and long straight tail; (**b**) contractile outer tail sheath.

Transmission electron microscopy was used to confirm phage presence and determine the morphology of LPJP1. Microscopy revealed the presence of a large icosahedral head, non-flexible long straight tail, and contractile outer tail sheath (Fig. 1a,b). These characteristics match the previously described myovirid morphotype of tailed phages (caudoviruses)^18,19^.

### Genome sequencing and engineering of LPJP1

**Figure 2:**
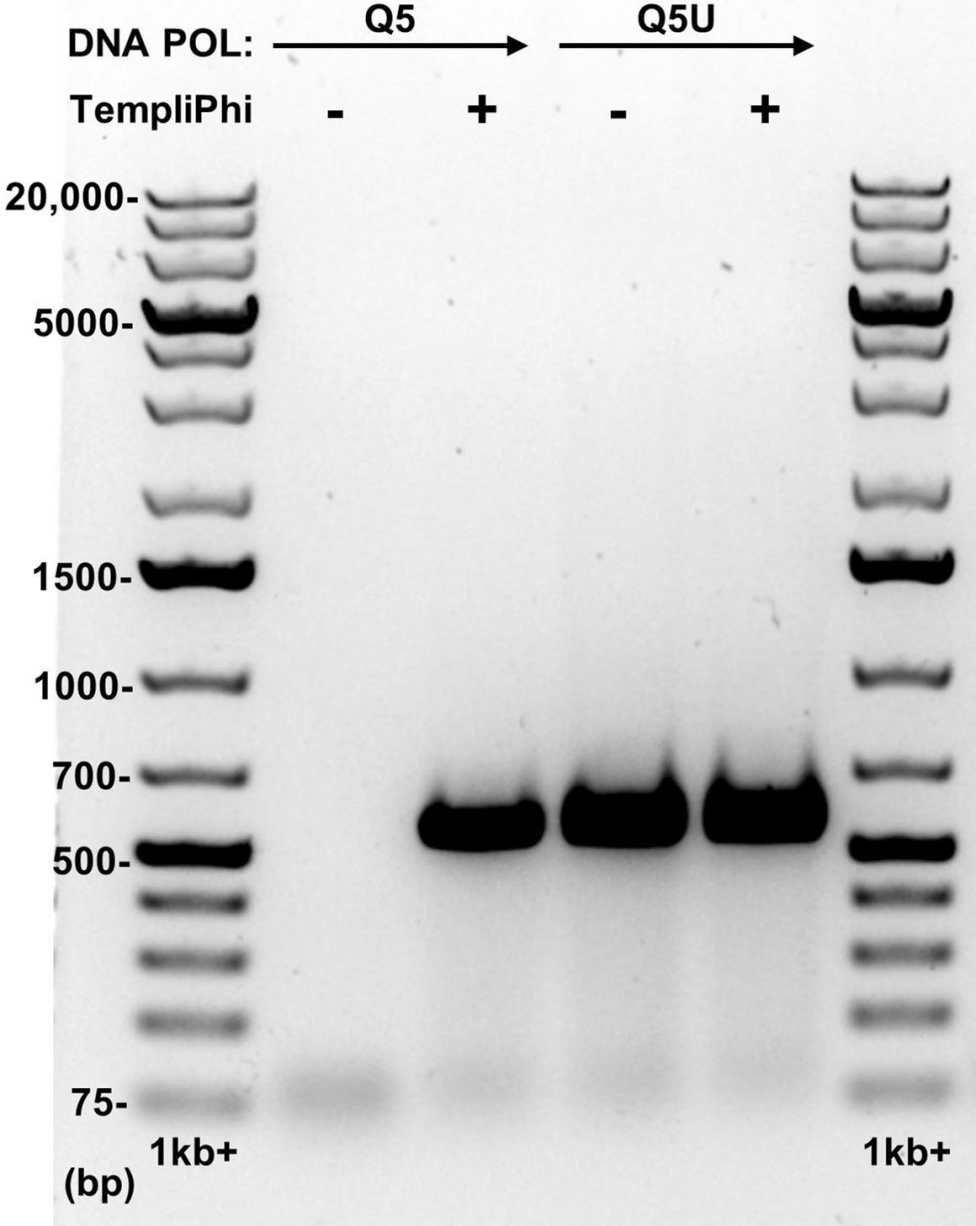
Gel electrophoresis of PCR-amplified LPJP1 DNA. Flanking lanes 1 and 6 contain O’GeneRuler 1 kb plus DNA ladder (Thermo Fisher Scientific, Waltham, MA, USA). Lanes 2 through 5 contain the PCR amplification of either native LPJP1 DNA (lanes 2 and 4) or TempliPhi-treated LPJP1 DNA (lanes 3 and 5) using either the Q5 DNA polymerase (lanes 2 and 3) or the uracil-tolerant Q5U DNA polymerase (lanes 4 and 5). Gel images were cropped to remove unrelated experimental data. Full-length gel image is presented in Supplementary Fig. 2.

The possibility of engineering the first *L. grayi* luciferase reporter phage from LPJP1 was intriguing. Previous success has been achieved by inserting a reporter downstream of a major capsid protein^10,12,15^. In order to replicate this approach with LPJP1, this location first had to be identified through sequencing. Surprisingly, initial attempts to sequence the genome of this phage were unsuccessful despite using a previously successful approach^10,12^. Despite obtaining sufficient DNA quantity and quality, library preparation using bead-lined transposomes (Illumina, San Diego, CA, USA) could not be achieved, failing at DNA amplification. This library preparation method is generally considered to be quite robust, demonstrating resistance to poor DNA quality and productivity with various DNA quantities^20^. One possible explanation for this failure is the presence of modified DNA base pairs, which have been observed to serve a number of functions in some bacteriophages^21^. In particular, the presence of deaminated base pairs, such as uracil or hypoxanthine, in DNA templates can inhibit commercial high-fidelity proof-reading archaeal polymerases^22,23^. Unfortunately, the polymerase used in the above library preparation kit remains undisclosed to our knowledge. To circumvent this possibility, DNA preparations of LPJP1 were amplified using the TempliPhi DNA amplification kit (Cytiva, Marlborough, MA, USA), which utilizes the Phi29 bacteriophage polymerase capable of reading past uracil-containing DNA^24,25^. PCR amplification is expected to convert uracil to thymine, as done typically with bisulfite-modified DNA^26^. As expected, library preparation was successfully completed using PCR-amplified DNA of LPJP1. Further support of both the presence of modified base pairs in LPJP1 and the ability to overcome this with the TempliPhi kit was obtained using the assembled sequence. Using this sequence, primers were designed to amplify approximately 550 bp of the LPJP1 genome within a predicted major capsid protein. PCR amplification was performed using equivalent amounts of either native LPJP1 DNA or TempliPhi-treated LPJP1 DNA. Additionally, the reaction was performed using either the Q5 polymerase or the uracil-tolerant Q5U polymerase (New England BioLabs, Ipswich, MA, USA). When these reactions were analyzed by gel electrophoresis, a distinct band of the expected size was observed with native DNA and the Q5U polymerase and TempliPhi-treated DNA with both polymerases (Fig. 2). A clear difference was observed between the uracil-sensitive Q5 polymerase and the uracil-tolerant Q5U polymerase on native phage DNA. Importantly, the failure of the Q5 polymerase in PCR was specific to native DNA and TempliPhi-treated DNA could be effectively amplified by both polymerases. These results support the hypothesis that LPJP1 contains modified base pairs and affirm the value of using an alternative DNA amplification method prior to traditional work flows if sequencing issues arise with novel phage.

Assembly of LPJP1 sequences revealed a large genome, 223,580 bp in length. This crosses the threshold of 200,000 bp to be considered a jumbo phage^27^. As reviewed previously, jumbo phages are rarely described and the vast majority (greater than 95%) of these phages recognize Gram-negative bacteria^28^. Among the few jumbo phages infecting Gram-positive bacteria, most recognize species within the genus *Bacillus*. Similar to previously described uracil-containing phages, the G + C content of LPJP1 was low (25.9%)^29^. Annotation of the assembled genome was performed, revealing 229 open reading frames (ORFs) and four tRNA genes. Unsurprisingly, the majority of ORFs encoded hypothetical proteins, which is in line with other recently annotated jumbo phages^30,31^. The sequence and annotation of LPJP1 was submitted to GenBank (accession number: MZ422438).

Manual examination of predicted ORFs in LPJP1 revealed a candidate major capsid protein (Supplementary Table 1). BLAST analysis of this protein sequence indicated homology with the precursor of the major head subunit protein of several Gram-positive jumbo phages. Top hits in this category were two recently sequenced *Staphylococcus* jumbo phage, MarsHill and Machias, followed by the *Bacillus* jumbo phage AR9. Amino acid homology was also observed with the structural protein sp46 precursor of both the *Bacillus* jumbo phage vB_BpuM-BpSP and the *Yersinia* jumbo phage phiR1-37. Sp46 has previously been identified as the probable major capsid protein in phiR1-37, strengthening the support for this prediction^32^. Homologous recombination was used then to mediate insertion of a late gene promoter and the NanoLuc luciferase sequence immediately downstream of the coding sequence for this candidate major capsid protein. Sequences used in engineering are provided (Supplementary Table 1). A recombinant NanoLuc-encoding LPJP1, referred hereafter as LPJP1.NL, was isolated, purified, and confirmed using genome sequencing.

### Limit of detection of LPJP1.NL

NanoLuc-encoding bacteriophages typically yield robust signal following infection of even a few bacterial cells^10,12,15^. In order to evaluate the sensitivity of LPJP1.NL for *L. grayi*, the limit of detection of this recombinant reporter was determined. LPJP1.NL was allowed to infect 0 to 10,000 colony forming units (CFU) of *L. grayi* for 4 h at 30 °C. After addition of substrate, NanoLuc production in each sample was quantified using a luminometer. Infection of *L. grayi* with LPJP1.NL yielded an easily detectable signal over medium background and increased proportionally with CFU (Table 1). Background from medium and reporter alone was a minimal 96 relative light units (RLU), while a single CFU of *L. grayi* was sufficient to achieve an average RLU signal of twice this amount. A threshold of approximately twice the background signal (190 RLU) was thus chosen to distinguish positive and negative samples. Using this cutoff, no positive signal was obtained in the absence of detectable CFU and all replicate wells were positive at burdens of 5 CFU or higher. As expected, variation between replicate wells and partial positives were noted when low bacterial burdens (1 or 2 CFU) were tested. Overall, these results indicate that LPJP1.NL is a sensitive reporter capable of producing a signal over background from a single CFU of *L. grayi* after a 4 h infection.

**Table 1:** Limit of detection of LPJP1.NL in *L. grayi*. Abbreviations: CFU = colony forming units, N = number of replicate wells, Avg. RLU = average relative light units, SD = standard deviation, CV = coefficient of variation, and Avg. S/B = average signal over background. Log phase cultures of *L. grayi* (ATCC 19,120) were diluted to the indicated burden and infected with LPJP1.NL for 4 h at 30 °C. Signal over background was calculated by dividing the Avg. RLU for each burden by the Avg. RLU of background (0 CFU). “Ratio of N Above Threshold” provides a ratio of replicates for each burden that generated a signal over 190 RLU, a threshold placed at approximately twice the Avg. RLU of background.

CFU	N	Avg. RLU	SD	% CV	Avg. S/B	Ratio of N Above Threshold
0	6	96	6	6	1.0	0/6
1	10	191	135	71	2.0	4/10
2	10	553	327	59	5.8	7/10
5	10	1012	474	47	10.6	10/10
10	10	2565	1055	41	26.9	10/10
100	10	24,056	2463	10	251.9	10/10
1000	6	264,210	24,637	9	2766.6	6/6
10,000	6	4,414,026	322,722	7	46,220.2	6/6

Previous concerns over the phenomenon of temperature-dependent plaque formation with *Listeria* phages and the implications on phage reporters have been raised^17^. Since LPJP1 shared this phenotype, an experiment was carried out to determine the role of temperature during infection on the signal production of LPJP1.NL. Minimal differences were noted between the RLU obtained from a 4 h infection at 30 °C or 37 °C and both temperatures yielded comparable results across all burdens (Supplementary Table 2). These results suggest that increased temperatures do not impede the production of signal from *Listeria* phage reporters.

### Inclusivity of LPJP1.NL detection

Based upon plaque formation and the limit of detection, LPJP1.NL was clearly capable of infecting the *L. grayi* subsp. *grayi* strain ATCC 19,120. However, it was not known whether or not this bacteriophage could broadly infect other *L. grayi* strains. Although the number of strains commercially available for testing is limited, four additional strains of *L. grayi* were obtained, including members of the only other subspecies *L. grayi* subsp. *murrayi*. Stationary phase cultures of each strain were diluted to a low burden (approximately 100 CFU) or a high burden (OD_600_ of 0.2) and infected with LPJP1.NL for 4 h. The cutoff of 190 RLU, approximately two times background, was used to distinguish between positive and negative detection for all inclusivity and exclusivity testing. Similar thresholds have proven useful in defining the host range of other NanoLuc bacteriophage reporters^9,10,12^. Using this method, all five *L. grayi* strains were successfully detected at both low and high burdens, indicating infection and subsequent NanoLuc production (Table 2). As expected, the use of stationary phase bacterial cells resulted in reduced signal compared to the limit of detection assay results for 100 CFU. Despite this decrease, LPJP1.NL was capable of infecting and detecting low burdens of all tested *L. grayi* strains, including representatives of both known subspecies.

**Table 2:** Inclusivity of LPJP1.NL detection. Abbreviations: ATCC = American Type Culture Collection CFU = colony forming units, RLU = relative light units, OD_600_ = optical density at 600 nm, Pos. = positive, and Neg. = negative. Strains were obtained from ATCC and the identification number of each strain is provided. The subspecies designation of *L. grayi* ATCC 700,545 is not known. Stationary phase cultures of each strain were diluted to the indicated CFU or OD_600_ prior to a 4 h infection at 30 °C with LPJP1.NL. Detection was evaluated for each sample using a positive threshold of 190 RLU, approximately twice medium background.

Bacteria	ATCC	Signal (RLU)	
		Low Burden (100 CFU)	High Burden (OD_600_ of 0.2)
*Listeria grayi* subsp. *grayi*	19,120	5,755 (Pos.)	274,905,152 (Pos.)
*Listeria grayi* subsp. *murrayi*	25,401	1,129 (Pos.)	76,794,888 (Pos.)
*Listeria grayi* subsp. *murrayi*	25,402	1,242 (Pos.)	278,885,856 (Pos.)
*Listeria grayi* subsp. *murrayi*	25,403	4,399 (Pos.)	176,249,632 (Pos.)
*Listeria grayi*	700,545	4743 (Pos.)	58,170,464 (Pos.)
Summary (Positives/Total Strains)		5/5	5/5

### Specificity of LPJP1.NL detection across other *Listeria* species

Other characterized *Listeria* phages of the myovirid morphotype, such as P100 and A511, are capable of infecting multiple *Listeria* species, dependent on serovar^33,34^. While LPJP1.NL had demonstrated coverage across several *L. grayi* strains, the ability of this phage to infect other *Listeria* species was of significant interest. An exclusivity panel of 150 *Listeria* strains was assembled, consisting of 13 species and 15 different serovars. Due to commercial availability and the public health importance of *L. monocytogenes*, 106 strains of this species were included. Of 66 *L. monocytogenes* with source-provided serovar information, 29 strains were serovar 1/2 (including fifteen 1/2a, nine 1/2b, and two 1/2c), five were serovar 3 (including two 3a, one 3b, and one 3c), and 32 were serovar 4 (including four 4a, nineteen 4b, one 4bx, three 4c, three 4d, and one 4e). In order to assess exclusivity, overnight stationary phase cultures of each strain were directly infected with LPJP1.NL for 4 h. This was expected to represent a significant bacterial burden and allow detection of even limited infection. Despite this burden, no strain in this *Listeria* panel yielded a positive result for NanoLuc production, as defined by the 190 RLU threshold (Table 3). RLU data and source of each strain are provided (Supplementary Table 3). The lack of any substantial signal over background from these strains suggests that LPJP1.NL is incapable of infecting other *Listeria* species besides *L. grayi*.

**Table 3:** Exclusivity of LPJP1.NL in non-*L. grayi Listeria* species. Stationary phase overnight cultures were infected for 4 h at 30 °C with LPJP1.NL. Positive detection was determined for each sample using a threshold of 190 relative light units (RLU). Individual RLU values and strain information are provided separately (Supplementary Table 3).

Bacteria	Positives/Total Strains
*Listeria aquatica*	0/1
*Listeria booriae*	0/1
*Listeria fleischmannii*	0/1
*Listeria floridensis*	0/1
*Listeria grandensis*	0/1
*Listeria innocua*	0/21
*Listeria ivanovii*	0/6
*Listeria marthii*	0/2
*Listeria monocytogenes*	0/106
*Listeria newyorkensis*	0/1
*Listeria riparia*	0/1
*Listeria seeligeri*	0/4
*Listeria welshimeri*	0/4
Summary	0/150

### Specificity of LPJP1.NL detection across other genera

To evaluate the specificity of LPJP1.NL for *Listeria*, an exclusivity panel of 26 Gram-negative and 19 Gram-positive strains was assembled. Representatives of 19 unique genera and 42 species were included. As done with *Listeria* species, overnight stationary phase cultures of each strain were infected with LPJP1.NL for 4 h. Unsurprisingly, no positive signal was detected from any of the 45 members of the exclusivity panel (Table 4). The absence of NanoLuc production in these strains further supports the notion that LPJP1.NL is highly specific for *L. grayi*.

**Table 4:** Exclusivity of LPJP1.NL in other genera (non-*Listeria*). Abbreviations: ATCC = American Type Culture Collection, RLU = relative light units, Pos. = positive, and Neg. = negative. Strains were obtained from ATCC and each strain’s identification number is provided. Stationary phase overnight cultures were infected for 4 h at 30 °C with LPJP1.NL. Detection was evaluated for each sample using a positive threshold of 190 RLU, approximately twice medium background.

Gram-Negative Bacteria	ATCC	RLU	Gram-Positive Bacteria	ATCC	RLU
*Acinetobacter baumannii*	19,606	48 (Neg.)	*Bacillus cereus*	14,579	48 (Neg.)
*Acinetobacter calcoaceticus*	23,055	95 (Neg.)	*Bacillus cereus*	13,061	108 (Neg.)
*Citrobacter braaki*	51,113	35 (Neg.)	*Bacillus circulans*	61	88 (Neg.)
*Citrobacter freundii*	8090	37 (Neg.)	*Bacillus coagulans*	7050	108 (Neg.)
*Citrobacter koseri*	25,408	81 (Neg.)	*Bacillus licheniformis*	9789	75 (Neg.)
*Cronobacter muytjensii*	51,329	69 (Neg.)	*Bacillus megaterium*	14,581	73 (Neg.)
*Cronobacter sakazakii*	12,868	65 (Neg.)	*Bacillus mycoides*	6462	65 (Neg.)
*Escherichia coli*	9637	48 (Neg.)	*Bacillus pumilus*	700,814	40 (Neg.)
*Escherichia fergusonii*	35,469	44 (Neg.)	*Bacillus subtilis*	23,857	49 (Neg.)
*Escherichia hermanii*	33,650	45 (Neg.)	*Bacillus subtilis*	6051	101 (Neg.)
*Edwardsiella tarda*	15,947	40 (Neg.)	*Bacillus weihenstephanensis*	12,826	55 (Neg.)
*Enterobacter cloacae*	13,047	31 (Neg.)	*Enterococcus faecalis*	19,433	87 (Neg.)
*Enterobacter kobei*	BAA-260	26 (Neg.)	*Enterococcus faecalis*	29,212	35 (Neg.)
*Hafnia alevi*	13,337	25 (Neg.)	*Enterococcus faecium*	19,434	74 (Neg.)
*Klebsiella aerogenes*	13,048	50 (Neg.)	*Lactobacillus plantarum*	14,917	68 (Neg.)
*Klebsiella oxytoca*	43,165	36 (Neg.)	*Lactobacillus rhamnosus*	7469	57 (Neg.)
*Klebsiella pneumoniae*	4352	42 (Neg.)	*Staphylococcus aureus*	27,660	37 (Neg.)
*Morganella morganii*	25,830	25 (Neg.)	*Staphylococcus epidermidis*	14,990	44 (Neg.)
*Pluralibacter gergovi*	33,028	33 (Neg.)	*Staphylococcus haemolyticus*	29,970	26 (Neg.)
*Proteus mirabilis*	43,071	14 (Neg.)			
*Proteus vulgaris*	33,420	30 (Neg.)			
*Pseudomonas aeruginosa*	27,853	70 (Neg.)			
*Serratia marcescens*	13,880	36 (Neg.)			
*Shigella flexneri*	12,022	45 (Neg.)			
*Shigella sonnei*	9290	24 (Neg.)			
*Yersinia enterocolitica*	23,715	51 (Neg.)			
Summary (Positives/Total Strains)		0/26	Summary (Positives/Total Strains)		0/19

## Discussion

*L. grayi* is one of several *Listeria* species often encountered in food-related environments^35–38^. Monitoring of *Listeria* species in these environments is often used by the food industry to identify and preemptively eliminate conditions supporting the growth of *L. monocytogenes*^5^. For these reasons, coverage of *L. grayi* is desirable and frequently incorporated into newly developed *Listeria* species detection assays^39–41^. Bacteriophage reporter assays represent one promising methodology capable of facilitating sensitive and specific detection of target organisms. Despite prior development of phage reporters for *Listeria*, phage-based detection of *L. grayi* has not been previously reported.

Although phages have been found for many *Listeria* species, phages targeting *L. grayi* have thus far remained elusive^6,33^. The absence of such phages prior to this study was both intriguing and potentially restrictive. As phage-based applications are often reliant on the engineering of naturally occurring phages, bacteria that cannot be infected by known phages are excluded from these otherwise promising technologies. In this study, the *L. grayi* phage LPJP1 was isolated from farm silage and formed clear plaques on lawns of *L. grayi* (Supplementary Fig. 1). Plaque formation only occurred at 30 °C and was not observed at 37 °C, a phenotype also observed in other *Listeria* phages (data not shown)^17^. TEM revealed that LPJP1 was a tailed phage (caudovirus) of the myovirid morphotype, with a large icosahedral head, a long straight tail, and a contractile outer tail sheath (Fig. 1a,b)^18,19^. At 223,580 base pairs, LPJP1 is, to our knowledge, the only *Listeria* phage to meet the “jumbo phage” designation. Annotation of LPJP1 revealed 229 ORFs, consisting largely of hypothetical proteins, and 4 tRNA genes. Analysis of predicted ORFs was conducted to identify a predicted major capsid protein (Supplementary Table 1). Homology was found between this identified protein and the major capsid proteins of other jumbo phages, particularly those infecting Gram-positive bacteria. LPJP1 thus appears to not only be the first *L. grayi* phage, but also the largest phage infecting the *Listeria* genus reported to date.

NanoLuc-encoding phages have repeatedly been found to exhibit impressive diagnostic sensitivity and robust signal production across multiple genera^9,10,12,13,15^. To our knowledge, however, no reporter has previously been generated from a jumbo phage. Despite this lack of precedent, a NanoLuc-encoding recombinant, LPJP1.NL was successfully generated using standard homologous recombination methods. Infection with LPJP1.NL produced substantial RLU from limited bacterial burdens without enrichment, requiring only a single log phase *L. grayi* bacterium to yield a detectable signal after a 4 h infection (Table 1). As expected, signal increased proportionally with bacterial burden. Sensitive detection of *L. grayi* by LPJP1.NL may have practical value as a member of a phage cocktail covering all *Listeria* species. Such a cocktail could facilitate phage-based monitoring of non-pathogenic species and allow for remediation of pro-*Listeria* environments prior to growth of pathogenic species. Additionally, the success of LPJP1.NL also highlights the untapped potential of jumbo phages in phage-based diagnostics as a whole.

Interestingly, comparable NanoLuc signal could be obtained from infections carried out at either 30 °C or 37 °C (Supplementary Table 2). Although plaques were not readily visualized at 37 °C, the robust signal from LPJP1.NL at this temperature suggests that phage DNA is injected successfully and expressed. Previous NanoLuc-encoding *Listeria* phage studies have also observed signal generation in strains that did not support plaque formation^15^. These observations demonstrate that some phage reporters may find success even in strains and conditions where visible plaque formation is not apparent. Although further studies are needed to elucidate the effects of temperature on this process, phage reporters such as LPJP1.NL may prove to be useful tools in understanding phage-host interactions in *Listeria*.

In addition to the parent *L. grayi* strain (ATCC 19,120), LPJP1.NL could mediate detection of low and high burdens of all five tested *L. grayi* strains in 4 h (Table 2). This panel was limited due to commercial availability of *L. grayi*, but included members of both subspecies, *L. grayi* subsp. *grayi* and *L. grayi* subsp. *murrayi*. Among these strains, detectable signal ranged from 1129 RLU to 5755 RLU with 100 stationary phase CFU to 58,170,464 to 278,885,856 RLU with higher bacterial burdens. Strain to strain differences, while minor, may be the result of several variables, including differences in growth rate, receptor density, or protein expression. LPJP1.NL was highly specific for *L. grayi*, demonstrating no cross-reactivity with other *Listeria* species or bacterial genera (Tables 3 and 4). The narrow host range of LPJP1 may indicate a cell receptor unique to *L. grayi*. Many *Listeria* phages are not species-dependent but are specific for one or more serovars^33^. Broad host range *Listeria* phages, such as A511, can infect multiple serovars by binding to rhamnose and N-acetylglucosamine, substituents of cell wall teichoic acids (WTAs)^42,43^. Unfortunately, *L. grayi* is rarely investigated, and to our knowledge the composition of the cell wall for these strains remains largely uncharacterized, including the presence of similar WTA decorations. Prior studies have noted that *L. grayi* is the only *Listeria* species with a homolog to TarM, a glycosyltransferase mediating WTA modification in *S. aureus*^44^. It is thus plausible that LPJP1 recognizes a WTA modification specific to *L. grayi*. Additionally, serological studies have revealed antigenic structures unique to *L. grayi*, which could participate in species-specific phage recognition^45^. Overall, the receptor and host range determinants of LPJP1 are unknown, but future studies may benefit from examination of WTA modifications or *L. grayi*-specific surface structures.

The sequencing issues encountered with LPJP1 may interfere with the discovery and engineering of similar jumbo phages. The success of library preparation only after DNA amplification with the phi29 bacteriophage polymerase suggests the presence of modified base pairs, such as uracil, in the genome of LPJP1. This hypothesis was further supported with PCR. In particular, DNA isolated from LPJP1 could only be directly amplified when an uracil-tolerant polymerase, such as Q5U, was used (Fig. 2). Importantly, modified base pairs are also found in several other Gram-positive jumbo phages^46–48^. In particular, the genomes of the *Bacillus* jumbo phages AR9 and PBS1 and the *Staphylococcus* jumbo phage S6 were all found to contain deoxyuridine in place of thymidine^46–48^. Alternative DNA base pairs are thought to primarily provide resistance against host defense systems, as DNA sequences containing modified base pairs are often resistant to various restriction enzymes^49,50^. Less commonly, however, modified base pairs have also been shown to contribute to other elements of phage biology, such as DNA packaging^51^. While the benefit of DNA amplification using an uracil-tolerant polymerase is clear, future studies will be necessary to confirm the presence, identity, and frequency of modified base pairs in LPJP1. The methods described in this study may thus be useful in facilitating the sequencing and engineering of other phages containing similar DNA modifications.

In this study, the isolation and engineering of a *L. grayi* phage, LPJP1, is described. NanoLuc-encoding recombinants were generated and found to be capable of sensitive and accurate detection of *L. grayi* in only 4 h. Practically, this reporter could prove to be a critical member of cocktails seeking to detect the presence of environmental *Listeria* species. Furthermore, this work highlights the value and diversity of naturally occurring phages as a foundation for genetic engineering and assay development. Using the methods described, the discovery and engineering of additional jumbo phages may prove beneficial in further expanding phage-based technologies.

## Methods

### Bacterial strains

Bacterial strains were obtained from the American Type Culture Collection (ATCC, Manassas, VA, USA), the Food Safety Laboratory of Cornell University (Ithaca, NY, USA), the University of Georgia (Athens, GA, USA) and Q Laboratories (Cincinnati, OH, USA). Cultures were routinely grown at 37 °C in brain heart infusion (BHI) broth (Becton Dickinson and Company, Sparks, MD, USA) with shaking at 225 revolutions per minute (rpm).

### Bacteriophage isolation, purification, and high titer stock preparation

The *L. grayi* bacteriophage LPJP1 was isolated from silage collected from a Wisconsin farm in the fall of 2018. Silage was provided by a Wisconsin farmer along with permission to use the material without restriction or limitation. Three g of silage was added to 30 mL of BHI media and allowed to diffuse at 2–8 °C for 72 h. To remove bacteria and debris, this solution was centrifuged at 4700×*g* for 10 min and the supernatant passed through a 0.45 µm polyethersulfone (PES) filter followed by a 0.2 µm PES filter (Cytiva, Marlborough, MA, USA). As an initial assessment to determine phage presence, a spot test was performed using a standard overlay method^52^. Briefly, 100 µL of a log phase culture of the *L. grayi* strain ATCC 19,120 was mixed with 3 mL of melted 0.5% (w/v) semi-solid BHI agar and then poured evenly atop BHI agar plates to form a lawn. Once the semi-solid agar solidified, plates were spotted with 5 µL of the filtered supernatant and incubated overnight at 30 °C. As spot testing yielded promising results, single plaques were isolated using a modification of the above overlay method. Log phase culture (100 µL), diluted filtered supernatant (100 µL), and melted semi-solid (3 mL) was poured atop a BHI agar plate and incubated overnight at 30 °C. Individual plaques were then picked and resuspended in 1 mL BHI media. Single plaque isolation, dilution, and plating was serially repeated five times.

High titer phage stocks were generated from broth lysates as described previously^10^. Briefly, a log phase culture of *L. grayi* (ATCC 19,120) was infected at a multiplicity of infection (MOI) of 0.05 and incubated statically for 5 min to facilitate bacteriophage adsorption. Infected cells were diluted into fresh BHI media and incubated at 30 °C with shaking at 225 rpm until lysis was visually apparent. To remove debris, the lysate was clarified by centrifugation. The supernatant was centrifuged to pellet phage and the pellet was resuspended in TMS Buffer (50 mM Tris–HCl, pH 7.8, 10 mM MgCl_2_, and 300 mM NaCl). Resuspended phage were treated with RNase and DNase I before being further purified using a sucrose density gradient (10–30%). The phages were resuspended in SM buffer (50 mM Tris–HCl, pH 7.5, 8 mM MgSO_4_·7H2O, 100 mM NaCl, and 0.01% (w/v) gelatin). This method yielded stock solutions with a titer of about 1.5 × 10^11^ plaque forming units (pfu) per mL.

### Bacteriophage characterization

To calculate the burst size and latent period of LPJP1, a standard one-step growth curve was performed using the *L. grayi* strain ATCC 19,120, as described previously^53^.

Transmission electron microscopy (TEM) of LPJP1 was performed as described previously^10^. Briefly, 400 mesh grids coated with a thin carbon film were glow discharged, floated on purified high titer phage stock, and subsequently stained with 2% uranyl acetate. A Tecnai G2 Spirit BioTWIN (FEI Company, Hillsboro, OR, USA) with an accelerating voltage set at 120 kV was used for image capture.

### Bacteriophage DNA isolation, sequencing, and PCR

To isolate phage DNA, 6 × 10^10^ pfu/mL were first heated at 95 °C for 2 min and then allowed to cool to room temperature. Initial heating is anticipated to induce ejection of capsid-contained DNA^54^. Sodium dodecyl sulfate (SDS), ethylenediaminetetraacetic acid (EDTA), and proteinase K were added to a final concentration of 0.1% SDS, 5 mM EDTA, and 53 µg/mL proteinase K. This mixture was incubated for 1 h at 50 °C before performing three rounds of phenol/chloroform extractions, serially separating the aqueous phase. After the addition of 0.1 volumes of 3 M sodium acetate and 2.5 volumes of ethanol to this phase, DNA was precipitated at −80 °C. Precipitated DNA was pelleted and washed twice with 70% ethanol. Washed DNA was pelleted once again, air dried, and finally re-suspended in 10 mM Tris–HCL, pH 8.0.

Phage DNA was quantified using absorbance at 260 nm on a NanoDrop (Thermo Fisher Scientific, Waltham, MA, USA) and sent to Laragen Inc. (Los Angeles, CA, USA) for sequencing. Laragen Inc. quantified dsDNA using a Qubit dsDNA HS assay kit (Thermo Fisher Scientific, Waltham, MA, USA) and performed library preparation using the Nextera DNA Flex library prep (Illumina, San Diego, CA, USA). When successful library preparation was achieved, samples underwent MiSeq whole genome sequencing (Illumina, San Diego, CA, USA) and contig assembly using SPAdes genome assembler app in Illumina’s BaseSpace. When indicated, DNA was amplified prior to sequencing using a Illustra TempliPhi DNA amplification kit (Cytiva, Marlborough, MA, USA) according to manufacturer’s instructions. Amplified DNA was quantified and submitted for sequencing, as described above. Phage annotation was performed using a database for *Caudovirales* phages available from the Millard Lab (http://millardlab.org/bioinformatics/lab_server/phage-genome-annotation/) and Prokka (v1.14.6)^55^. The annotated genome was deposited in GenBank (accession number: MZ422438).

Polymerase chain reaction (PCR) was performed using either the Q5 hot start high-fidelity DNA polymerase or the Q5U hot start high-fidelity DNA polymerase (New England BioLabs, Ipswich, MA, USA). DNA templates were either native phage DNA or TempliPhi-amplified DNA prepared as described above. To ensure elements of the TempliPhi reaction did not interfere with PCR, DNA was purified using a Monarch PCR and DNA cleanup kit (New England BioLabs, Ipswich, MA, USA). PCR was performed using 2 ng of DNA, either amplified or native, and either the Q5 or Q5U polymerase, as per manufacturer’s instructions. Primers were (5′-AGGAAACAGCTATGACATGATTACGTTATCATAAAACTTTCGATGTAC-3′) and (5′-ATTTATACTCTATTTAACGAGCGTATTGAGTTG-3′) and were obtained from Eurofins Genomics (Louisville, KY, USA). PCR reactions were assessed using gel electrophoresis. One-half of each reaction was mixed with GelPilot DNA loading dye (Qiagen, Hilden, Germany) and loaded onto a 1% agarose gel (Bio-Rad Laboratories, Hercules, CA, USA) in standard Tris–acetate-EDTA (TAE) buffer. For size comparison, O’GeneRuler 1 kb plus DNA ladder (Thermo Fisher Scientific, Waltham, MA, USA) was added to flanking wells. SYBR Safe DNA Gel Strain (Invitrogen, Carlsbad, CA, USA) was included in the gel to allow visualization of results. The gel was electrophoresed for 100 Vh while submerged in TAE buffer. Gel imaging was performed using the Gel Doc EZ system and ImageLab (version 6.0.1) software (Bio-Rad, Hercules, CA, USA).

### Design and engineering of luciferase reporter phage recombinants

Homologous recombination was used to insert a late gene promoter and NanoLuc downstream of the predicted major capsid protein to create a NanoLuc-encoding recombinant of LPJP1. No predicted genes are anticipated to be disrupted following recombination at this site. A similar approach has proven successful in other bacteriophages and previously yielded promising phage reporters^10,12^. A predicted major capsid protein was identified in LPJP1 by manual screening of possible open reading frames in the LPJP1 genome assembly (Supplementary Table 1). This prediction was supported by homology observed using BLAST (BLASTP)^56,57^. A shuttle vector containing homology flanks to mediate recombination, a late gene promoter, and the NanoLuc sequence was designed as follows. The upstream flank begins with a KpnI restriction site followed by 500 bp of homology upstream of the insertion site. The insertion site was immediately after the predicted major capsid protein of LPJP1. Following this flank, a promoter was added containing −10 and −35 elements from A511’s major capsid protein (a late gene) sequence and an A511 consensus ribosome binding site sequence^42,58^. The NanoLuc coding sequence was added following this promoter. The downstream flank contained 500 bp of homology downstream of the target insertion site and was followed by a SalI restriction site. Sequences for both flanks and the promoter are provided (Supplementary Table 1). The sequence of NanoLuc has been previously described^59^. These sequences were synthesized and inserted between the KpnI and SalI restriction sites of the multiple cloning site in the previously described shuttle vector pCE104^60^. All cloning was performed by Blue Heron Biotech (Bothell, WA, USA).

ATCC 19,120, the *L. grayi* strain used during isolation of LPJP1, was also selected as a host for recombination. Electrocompetent ATCC 19,120 was generated using a modification of a previously described method^61^. 10 µg/mL of Penicillin G (Thermo Fisher Scientific, Waltham, MA, USA) was added to a log phase culture of ATCC 19,120 and incubated for 1 h at 37 °C with shaking (225 rpm). Treated cultures were then chilled on ice for 30 min, pelleted, and washed three times with cold SMP (272 mM sucrose, 1 mM MgCl_2_, 7 mM sodium phosphate pH 6.8). Cells were pelleted a final time and resuspended in 1 mL of cold SMP. 100 µL aliquots were frozen and stored at −80 °C until use. 100 ng of homologous recombination plasmid DNA was added to thawed electrocompetent cells, incubated on ice for 15 min, and transferred to a 0.2 cm cuvette. Electroporation was carried out using a MicroPulser electroporation apparatus (Bio-Rad Laboratories, Hercules, CA, USA) with a voltage of 2.5 kV and a 2.8 ms time constant. Electroporated cells were recovered in fresh BHI for 2 h at 30 °C before being plated on BHI agar plates containing 5 µg/mL erythromycin sulfate (Alfa Aesar, Haverhill, MA, USA). Plates were incubated at 37 °C for 24 h before being examined for transformants.

Erythromycin-resistant colonies were picked, grown up in BHI containing 5 µg/mL erythromycin sulfate, and infected with varying amounts of LPJP1 equivalent to MOIs of 0.01 to 0.1. Infections were incubated for 4.5 h at 30 °C with 225 rpm shaking. To remove debris and intact bacteria, samples were centrifuged for 2 min at 6800×*g* and the supernatant passed through a 0.45 µm PES filter. This solution was transferred to a 100 kDa pore protein concentrator PES column (Pierce Biotechnology, Rockford, IL) and buffer exchanged with TMS. Potential phage recombinants were identified from this reaction as previously described^10,12^. Briefly, enrichment of limiting dilutions followed by screening for luciferase production was used to improve the likelihood of finding a recombination event. When found to be positive, these enrichments were plated to isolate single plaques on semi-solid agar, as described above. Candidate plaques were picked, mixed with diluted cultures of ATCC 19,120, and evaluated for NanoLuc production. Single plaque isolation, dilution, and plating was serially performed five times to establish a pure NanoLuc-encoding recombinant, called LPJP1.NL. Using the same preparation method described above, high titer stocks of LPJP1.NL were made and sent for genome sequencing. Analysis of the assembled genome of LPJP1.NL verified that the homologous recombination occurred as desired, inserting the promoter and NanoLuc downstream of the predicted major capsid protein.

### Limit of detection of LPJP1.NL

The limit of detection of LPJP1.NL in *L. grayi* was determined as follows. A log phase culture (OD_600_ of 0.1 to 0.5) of the *L. grayi* strain ATCC 19,120 was diluted in BHI media to achieve 10 to 100,000 CFU/mL. 100 µL of each bacterial dilution or BHI media was added to at least six replicate wells on a 96-well plate. Each well was infected with 10 µL of LPJP1.NL at 1.2 × 10^7^ pfu/mL and the infection was allowed to proceed for 4 h at 30 °C. At the end of the infection, 65 µL of a luciferase detection solution was added to each well. The luciferase detection solution consisted of 50 µL of NanoGlo buffer, 15 µL of Renilla lysis buffer, and 1 µL of NanoGlo substrate (Promega Corp., Madison, WI, USA). A GloMax Navigator luminometer (Promega Corp., Madison, WI, USA), set to a 3 min wait time and 1 s integration, was used to detect light production. Using this method, relative light units (RLU) values were obtained for each well and averaged among replicates.

When indicated, the above protocol was modified to determine the effect of infection temperature on signal production. Bacteria were prepared and infected as described above with the following exception. For each burden, two wells were infected with LPJP1.NL. One set of wells was infected for 4 h at 30 °C, while the other set was infected for 4 h at 37 °C. RLU values were obtained using a luciferase detection solution as described above.

### Determining the host range of LPJP1.NL

The inclusivity of LPJP1.NL was determined using several commercially available *L. grayi* strains. Overnight stationary phase cultures of each strain were diluted in BHI media to either a high burden (OD_600_ of 0.2) or a low burden (1000 CFU/mL). 100 µL of each dilution was added to a well of a 96-well plate and infected with 10 µL of LPJP1.NL at 1.2 × 10^7^ pfu/mL. As done previously, infected wells were incubated for 4 h at 30 °C before the addition of 65 µL of luciferase detection solution. RLU values were obtained for each sample on the GloMax Navigator as described above. Samples were evaluated using a threshold of 190 RLU, which was approximately twice the signal over background (BHI media control).

The exclusivity of LPJP1.NL was determined using both other *Listeria* species and a multitude of Gram-positive and Gram-negative genera. To assess the possibility of even minute signal production, a high bacterial burden was selected for testing. To this end, 100 µL of overnight culture was directly added to a 96-well plate and infected with 10 µL of LPJP1.NL at 1.2 × 10^7^ pfu/mL. As done previously, infected wells were incubated for 4 h at 30 °C before the addition of 65 µL of luciferase detection solution. Luminescence was measured and analyzed as described above for inclusivity assays.

## Supplementary Information

Supplementary Information.

## Data Availability

Data supporting the reported results can be found in the manuscript and supplementary materials with the following exception. Sequence and annotation of LPJP1 was deposited to GenBank (accession number: MZ422438). Researchers receiving resources generated in this study may be asked to sign a Materials Transfer Agreement that covers potential commercial use.
