# Supplementary material for: Isolation and engineering of a Listeria grayi bacteriophage
Source: Sci Rep. 2021 Sep 23;11:18947. doi: 10.1038/s41598-021-98134-1 (PMC8460666; doi:10.1038/s41598-021-98134-1)
Supplement: Supplementary file 1 — Supplementary Information. [file 41598_2021_98134_MOESM1_ESM.docx]

**Supplementary Information**

**Title:** “Isolation and Engineering of a *Listeria grayi* Bacteriophage”

**Authors:** Stephen Erickson ^1,*^, John Paulson ^1^, Matthew Brown ^2^, Wendy Hahn ^1^, Jose Gil ^3^, Rocío Barron-Montenegro ^4,5^, Andrea I. Moreno-Switt ^4,5^, Marcia Eisenberg ^2^ and Minh M. Nguyen ^1^

^1^Laboratory Corporation of America Holdings, New Brighton, MN 55112

^2^Laboratory Corporation of America Holdings, Burlington, NC 27215

^3^Laboratory Corporation of America Holdings, Los Angeles, CA 90062

^4^Escuela de Medicina Veterinaria, Facultad de Agronomía e Ingeniería Forestal, Facultad de Ciencias Biológicas, Facultad de Medicina, Pontificia Universidad Católica de Chile, Santiago, Chile

^5^Millennium Initiative for Collaborative Research on Bacteria Resistance (MICROB-R), Santiago, Chile

^*^e-mail: erickss@labcorp.com


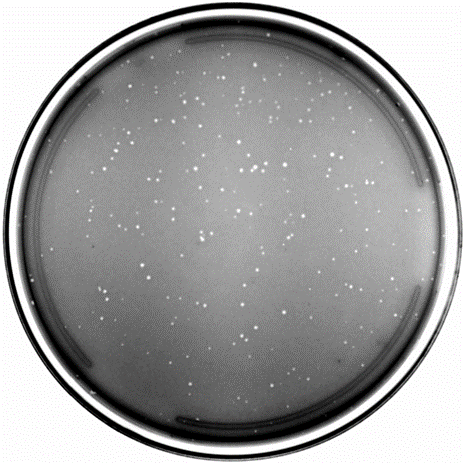


**Supplementary Figure 1.** Plaque formation by LPJP1 on *L. grayi* (ATCC 19120). LPJP1 was diluted, mixed with overnight culture and 0.5% semi-solid BHI agar, and poured evenly atop BHI agar plates. Plates were imaged after overnight incubation at 30 °C.


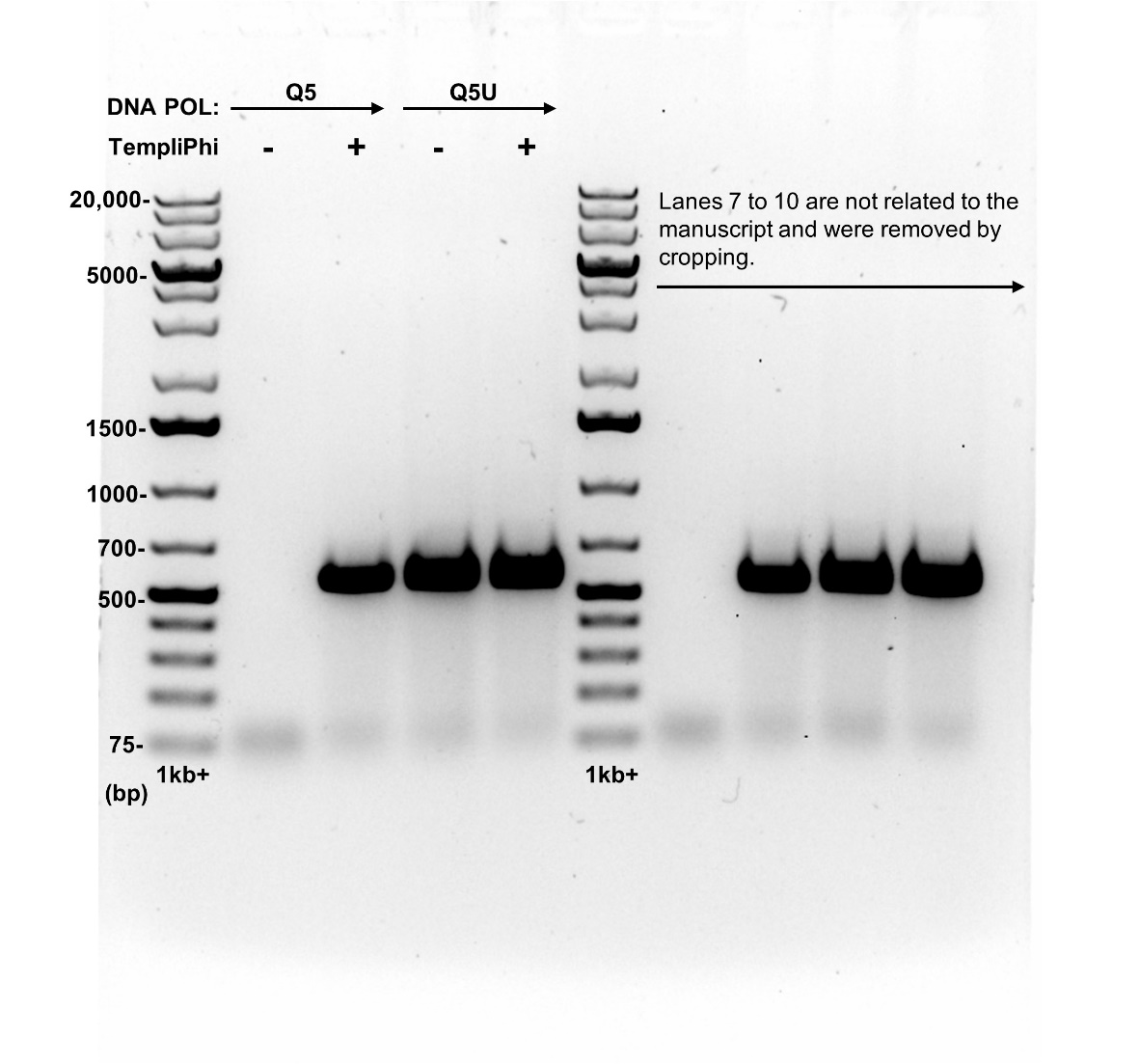


**Supplementary Figure 2.** Gel electrophoresis of PCR-amplified LPJP1 DNA. Annotated full-length gel image of Fig. 2. Lanes 1 and 6 contain O’GeneRuler 1kb plus DNA ladder. Lanes 2 through 5 contain the PCR amplification of either native LPJP1 DNA (lanes 2 and 4) or TempliPhi-treated LPJP1 DNA (lanes 3 and 5) using either the Q5 DNA polymerase (lanes 2 and 3) or the uracil-tolerant Q5U DNA polymerase (lanes 4 and 5). Lanes 7 to 10 are not related to the manuscript and were removed by cropping for the main text.

| **Feature** | **DNA or Amino Acid Sequence** |
| --- | --- |
| Upstream  HR Flank | GGTACCATGTACATCCAAATGGTGGATACAACATGCATCCGCATGAATGGCGCCGTGGTATTCGTGACGTTATTGACTGGATGTCTCAAGCAATGAAAAATGATTACAAAACTTATGATGCTTACTTTGTAATTGTTGGTAATCCAATTGACACTCAATTGATTCCTGACATTGAATGGGAATTCCAAGGAGCTACTGATGAAGTTGCGGGAATTAACGTTTCTTATAGCGTTGGTGCTTCTTCTACAGTTAACCGTTATAAAGTTGTTTCTTCTGACTTAGTACCTGCTGGAGATCTATTAATCTTTGCAGTTCCTACTCGTGAAGACTTCAAAACTTACGAATACTACCCATACACTTTCAATATCGTTAATAATTACAACAATGCTGTTAACCAAAGCGTTCCTAACATTATGCTTTCTCGCCGTTATACAGTTGAAGAATTTGTTCCAATTATCGGTAAAGTTACAATTAAAAACAACGATGCAACTCAATACGCTCGTTAA |
| Downstream HR Flank | TAAATATTATGAACCTTTCAGAGACCTTTTGGTTTCTGAAAGGTTATTATTTATAAAAATTTAAATTGGCGGTGCAACCATGCATATTGATTATGGTATTGAAAAGAATATATCAAAGTACTTACATGAAGAATTACTAACTGAAGATATCTATAATCATCCTTTATTAAAAAAGATTGATGATGAATTTCAGAAAATATTAGATGAAGATAATATTAATGATACTAAACTACCAGTAACATCATATAAAAAAATTCAAAATATAATTAAATATGTCTCAACTATATTTAATATAAATTTAATTATAACGATTGATAACGATAATATCCTTACTTATGGAATGATGACATTTATTCCGGTTAAAAATCTAACAAAGATATCTAATAATATAAAGAAGATTGTACTTCAACCAAAAACTGGTTTTGAATATATTAAAACTGAAGTTATTGAAATTAAAATACAGAAAAAATTAATATCTTTCATTAAGGATCCAAACTTACGTCGAC |
| Promoter | ATAGAGTATAAATTCTATTCAGTTTCGTATATTAATACTAGTAGATAGGAGGTGTTTCGA |
| Predicted Major Capsid Protein | MRNFKRITKTNTDSFSTHLSETQDYFANTKGTNITGQDIAAIIVNEQYFDEYATRLLEGFDADLSEELGVLLENTRSNIMESLGGITPFASLSMPVLVKLWARLSMVNAIPTTPVTTPAFVVPTIKPYTIGPDGEKYYLPEAINTIPEHFVSLRQLKEDITITGGRLSDYDLFTGVSTADRAKGDQVDRKFQIVAGTWSDSYNVADAALGEYELKGQALKMDIHGNIFGKVTYTTDGNGATNEDTIMGHVDVEKGRLDLTSLSGKLTEFKIQGFVSSEMHTGTTQVGFDVDDRTINIGTAPHIEGILPIESVQDSKAMYDIDAAAVIVDTMSATSAQKVDTDLIEFLLRSYEGTNAAYHKTFDVHPNGGYNMHPHEWRRGIRDVIDWMSQAMKNDYKTYDAYFVIVGNPIDTQLIPDIEWEFQGATDEVAGINVSYSVGASSTVNRYKVVSSDLVPAGDLLIFAVPTREDFKTYEYYPYTFNIVNNYNNAVNQSVPNIMLSRRYTVEEFVPIIGKVTIKNNDATQYAR* |

**Supplementary Table 1:** Sequences used in homologous recombination (HR). Upstream HR flank consists of a KpnI restriction site (underlined) and 500 bp of homology upstream of desired insertion site (immediately following the predicted major capsid protein). Downstream HR flank consists of 500 bp of homology downstream of desired insertion site, followed by a SalI restriction site (underlined). Key elements of the promoter sequence are underlined, including the -35 (ATAGAGTA) and -10 (TATATT) elements and the ribosome binding site (AGGAGGTG). Predicted major capsid protein of LPJP1 was identified by manual examination of open reading frames.

| **CFU** | **RLU 30 °C** | **RLU 37 °C** |
| --- | --- | --- |
| 0 | 89 (Neg.) | 88 (Neg.) |
| 1 | 248 (Pos.) | 909 (Pos.) |
| 2 | 821 (Pos.) | 966 (Pos.) |
| 5 | 1,692 (Pos.) | 2,926 (Pos.) |
| 10 | 2,155 (Pos.) | 2,893 (Pos.) |
| 100 | 24,407 (Pos.) | 34,156 (Pos.) |
| 1,000 | 250,905 (Pos.) | 391,186 (Pos.) |
| 10,000 | 5,940,865 (Pos.) | 5,546,562 (Pos.) |

**Supplementary Table 2:** Comparison of limit of detection after 30 °C or 37 °C infection. Abbreviations: CFU = colony forming units, RLU = relative light units, Pos. = positive, and Neg. = negative. Log phase cultures of *L. grayi* (ATCC 19120) were diluted to the indicated burden and infected with LPJP1.NL for 4 h at 30 °C or 37 °C. Detection was determined for each sample using a positive threshold of 190 RLU, approximately twice medium background.

| **Bacteria** | **Strain** | **Source** | **Serovar** | **RLU** |
| --- | --- | --- | --- | --- |
| *Listeria aquatica* | FSL S10-1188 | FSL |  | 64 |
| *Listeria booriae* | FSL A5-0281 | FSL |  | 125 |
| *Listeria fleischmannii* | FSL F6-1016 | FSL |  | 98 |
| *Listeria floridensis* | FSL S10-1187 | FSL |  | 166 |
| *Listeria grandensis* | FSL F6-0971 | FSL |  | 67 |
| *Listeria innocua* | 33090 | ATCC | 6a | 69 |
| *Listeria innocua* | BAA-680 | ATCC | 6a | 49 |
| *Listeria innocua* | 33091 | ATCC | 6b | 78 |
| *Listeria innocua* | 43547 | ATCC | 6b | 84 |
| *Listeria innocua* | 51742 | ATCC |  | 50 |
| *Listeria innocua* | BAA-349 | ATCC |  | 37 |
| *Listeria innocua* | 9 | UGA |  | 73 |
| *Listeria innocua* | 15 | UGA |  | 61 |
| *Listeria innocua* | 16 | UGA |  | 69 |
| *Listeria innocua* | 18 | UGA |  | 54 |
| *Listeria innocua* | 72 | UGA |  | 56 |
| *Listeria innocua* | 80 | UGA |  | 60 |
| *Listeria innocua* | 92 | UGA |  | 81 |
| *Listeria innocua* | Silliker 9 | UGA |  | 90 |
| *Listeria innocua* | Silliker 9 NAR | UGA |  | 85 |
| *Listeria innocua* | Silliker 16 | UGA |  | 61 |
| *Listeria innocua* | Silliker 16 NAR | UGA |  | 58 |
| *Listeria innocua* | Silliker 23 NAR | UGA |  | 90 |
| *Listeria innocua* | Silliker 24 | UGA |  | 63 |
| *Listeria innocua* | Silliker 24 NAR | UGA |  | 72 |
| *Listeria innocua* | Silliker106 NAR | UGA |  | 56 |
| *Listeria ivanovii* | 19119 | ATCC |  | 80 |
| *Listeria ivanovii* | 49953 | ATCC |  | 87 |
| *Listeria ivanovii* | BAA-678 | ATCC | 5 | 92 |
| *Listeria ivanovii* | BAA-753 | ATCC |  | 65 |
| *Listeria ivanovii* | 700402 | ATCC |  | 66 |
| *Listeria ivanovii* | 49954 | ATCC |  | 59 |
| *Listeria marthii* | FSL S4-120 | FSL |  | 58 |
| *Listeria marthii* | FSL S4-965 | FSL |  | 54 |
| *Listeria monocytogenes* | 19111 | ATCC | 1/2a | 35 |
| *Listeria monocytogenes* | 51772 | ATCC | 1/2a | 67 |
| *Listeria monocytogenes* | 51774 | ATCC | 1/2a | 71 |
| *Listeria monocytogenes* | 51775 | ATCC | 1/2a | 77 |
| *Listeria monocytogenes* | BAA-679 | ATCC | 1/2a | 70 |
| *Listeria monocytogenes* | BAA-2657 | ATCC | 1/2a | 52 |
| *Listeria monocytogenes* | BAA-2659 | ATCC | 1/2a | 55 |
| *Listeria monocytogenes* | BAA-2660 | ATCC | 1/2a | 74 |
| *Listeria monocytogenes* | F8369 | UGA | 1/2a | 60 |
| *Listeria monocytogenes* | FSL J2-020 | FSL | 1/2a | 53 |
| *Listeria monocytogenes* | FSL J2-066 | FSL | 1/2a | 65 |
| *Listeria monocytogenes* | FSL C1-056 | FSL | 1/2a | 57 |
| *Listeria monocytogenes* | FSL J2-031 | FSL | 1/2a | 34 |
| *Listeria monocytogenes* | FSL J2-054 | FSL | 1/2a | 50 |
| *Listeria monocytogenes* | FSL J2-063 | FSL | 1/2a | 51 |
| *Listeria monocytogenes* | BAA-751 | ATCC | 1/2b | 66 |
| *Listeria monocytogenes* | BAA-839 | ATCC | 1/2b | 73 |
| *Listeria monocytogenes* | 51780 | ATCC | 1/2b | 71 |
| *Listeria monocytogenes* | BAA-2658 | ATCC | 1/2b | 58 |
| *Listeria monocytogenes* | FSL J2-064 | FSL | 1/2b | 58 |
| *Listeria monocytogenes* | FSL J1-177 | FSL | 1/2b | 70 |
| *Listeria monocytogenes* | FSL J2-035 | FSL | 1/2b | 72 |
| *Listeria monocytogenes* | G6006 | UGA | 1/2b | 56 |
| *Listeria monocytogenes* | F8255 | UGA | 1/2b | 43 |
| *Listeria monocytogenes* | 51779 | ATCC | 1/2c | 51 |
| *Listeria monocytogenes* | FSL J1-094 | FSL | 1/2c | 48 |
| *Listeria monocytogenes* | 984 | ATCC | 1 | 46 |
| *Listeria monocytogenes* | 9525 | ATCC | 2 | 102 |
| *Listeria monocytogenes* | 19112 | ATCC | 2 | 38 |
| *Listeria monocytogenes* | 19113 | ATCC | 3 | 160 |
| *Listeria monocytogenes* | 51782 | ATCC | 3a | 89 |
| *Listeria monocytogenes* | FSL C1-115 | FSL | 3a | 56 |
| *Listeria monocytogenes* | FSL J1-169 | FSL | 3b | 55 |
| *Listeria monocytogenes* | FSL J1-049 | FSL | 3c | 45 |
| *Listeria monocytogenes* | 51781 | ATCC | 4 | 45 |
| *Listeria monocytogenes* | 19114 | ATCC | 4a | 55 |
| *Listeria monocytogenes* | FSL J1-031 | FSL | 4a | 67 |
| *Listeria monocytogenes* | FSL J1-168 | FSL | 4a | 67 |
| *Listeria monocytogenes* | FSL W1-112 | FSL | 4a | 62 |
| *Listeria monocytogenes* | 19115 | ATCC | 4b | 46 |
| *Listeria monocytogenes* | 13932 | ATCC | 4b | 55 |
| *Listeria monocytogenes* | 51776 | ATCC | 4b | 53 |
| *Listeria monocytogenes* | 51777 | ATCC | 4b | 76 |
| *Listeria monocytogenes* | 51778 | ATCC | 4b | 92 |
| *Listeria monocytogenes* | FSL J1-108 | FSL | 4b | 51 |
| *Listeria monocytogenes* | FSL J1-110 | FSL | 4b | 55 |
| *Listeria monocytogenes* | FSL J1-116 | FSL | 4b | 75 |
| *Listeria monocytogenes* | FSL J1-225 | FSL | 4b | 46 |
| *Listeria monocytogenes* | FSL J1-126 | FSL | 4b | 73 |
| *Listeria monocytogenes* | FSL J1-158 | FSL | 4b | 37 |
| *Listeria monocytogenes* | FSL C1-122 | FSL | 4b | 64 |
| *Listeria monocytogenes* | F2365 | UGA | 4b | 45 |
| *Listeria monocytogenes* | F2365-0770 | UGA | 4b | 38 |
| *Listeria monocytogenes* | F8027 | UGA | 4b | 48 |
| *Listeria monocytogenes* | G1091 | UGA | 4b | 74 |
| *Listeria monocytogenes* | FSL N1-225 | FSL | 4b | 53 |
| *Listeria monocytogenes* | FSL N1-227 | FSL | 4b | 86 |
| *Listeria monocytogenes* | FSL R2-500 | FSL | 4b | 72 |
| *Listeria monocytogenes* | FSL J1-129 | FSL | 4bx | 69 |
| *Listeria monocytogenes* | FSL W1-110 | FSL | 4c | 87 |
| *Listeria monocytogenes* | FSL W1-111 | FSL | 4c | 94 |
| *Listeria monocytogenes* | 19116 | ATCC | 4c | 30 |
| *Listeria monocytogenes* | 19117 | ATCC | 4d | 49 |
| *Listeria monocytogenes* | Li 2108 | UG | 4d | 89 |
| *Listeria monocytogenes* | FSL J1-107 | FSL | 4d | 58 |
| *Listeria monocytogenes* | 19118 | ATCC | 4e | 51 |
| *Listeria monocytogenes* | 23073 | ATCC |  | 44 |
| *Listeria monocytogenes* | 23074 | ATCC |  | 41 |
| *Listeria monocytogenes* | 7302 | ATCC |  | 74 |
| *Listeria monocytogenes* | 7644 | ATCC |  | 79 |
| *Listeria monocytogenes* | 43256 | ATCC |  | 64 |
| *Listeria monocytogenes* | 15313 | ATCC |  | 62 |
| *Listeria monocytogenes* | 35152 | ATCC |  | 26 |
| *Listeria monocytogenes* | CWD 1554 | Q Labs |  | 51 |
| *Listeria monocytogenes* | H7557 | UGA |  | 52 |
| *Listeria monocytogenes* | H7738 | UGA |  | 52 |
| *Listeria monocytogenes* | FSL M1-004 | FSL |  | 51 |
| *Listeria monocytogenes* | Bilmar | UGA |  | 28 |
| *Listeria monocytogenes* | Brie | UGA |  | 47 |
| *Listeria monocytogenes* | Coleslaw | UGA |  | 49 |
| *Listeria monocytogenes* | Jalisco | UGA |  | 51 |
| *Listeria monocytogenes* | Jalisco GZ7 | UGA |  | 71 |
| *Listeria monocytogenes* | Silliker 15 | UGA |  | 62 |
| *Listeria monocytogenes* | Silliker 15 NAR | UGA |  | 62 |
| *Listeria monocytogenes* | Silliker 17 | UGA |  | 63 |
| *Listeria monocytogenes* | Silliker 17 NAR | UGA |  | 76 |
| *Listeria monocytogenes* | Silliker 18 | UGA |  | 48 |
| *Listeria monocytogenes* | Silliker 18 NAR | UGA |  | 61 |
| *Listeria monocytogenes* | Silliker 32 NAR | UGA |  | 135 |
| *Listeria monocytogenes* | Silliker 70 | UGA |  | 52 |
| *Listeria monocytogenes* | Silliker 70 NAR | UGA |  | 67 |
| *Listeria monocytogenes* | Silliker 72 NAR | UGA |  | 73 |
| *Listeria monocytogenes* | Silliker 80 NAR | UGA |  | 50 |
| *Listeria monocytogenes* | Silliker 106 | UGA |  | 35 |
| *Listeria monocytogenes* | 2011L-2663 | UGA |  | 60 |
| *Listeria monocytogenes* | 2011L-2626 | UGA |  | 65 |
| *Listeria monocytogenes* | 2011L-2625 | UGA |  | 121 |
| *Listeria monocytogenes* | 2011L-2624 | UGA |  | 164 |
| *Listeria monocytogenes* | F6900 | UGA |  | 43 |
| *Listeria monocytogenes* | SLR 516 | UGA |  | 85 |
| *Listeria monocytogenes* | G3990 | UGA |  | 54 |
| *Listeria monocytogenes* | LCDC 81-861 | UGA |  | 72 |
| *Listeria monocytogenes* | 101M | UGA |  | 48 |
| *Listeria monocytogenes* | 108 M | UGA |  | 50 |
| *Listeria monocytogenes* | V7 | UGA |  | 106 |
| *Listeria monocytogenes* | 1A1 | UGA |  | 101 |
| *Listeria newyorkensis* | FSL M6-0635 | FSL |  | 79 |
| *Listeria riparia* | FSL S10-1204 | FSL |  | 65 |
| *Listeria seeligeri* | 35967 | ATCC |  | 82 |
| *Listeria seeligeri* | 51334 | ATCC |  | 65 |
| *Listeria seeligeri* | 51335 | ATCC | 4a | 45 |
| *Listeria seeligeri* | 2/25/04 | UGA |  | 59 |
| *Listeria welshimeri* | 35897 | ATCC | 6b | 81 |
| *Listeria welshimeri* | 43549 | ATCC | 6b | 74 |
| *Listeria welshimeri* | 43551 | ATCC | 6a | 69 |
| *Listeria welshimeri* | 43550 | ATCC | 1/2b | 81 |

**Supplementary Table 3:** RLU for exclusivity of LPJP1.NL in non-*L. grayi* *Listeria* species. Abbreviations: RLU = relative light units. Strains were obtained from either the American Type Culture Collection (ATCC, Manassas, VA, USA), the Food Safety Laboratory of Cornell University (FSL, Ithaca, NY, USA), the University of Georgia (UGA, Athens, GA, USA), or Q Laboratories (Q Labs, Cincinnati, OH, USA). Serovar of each strain is indicated when provided by source. RLU values are analyzed and summarized in Table 3 of the main text.
